# Recent Advances in the Stereoselective Polymerization of Epoxides and Applications of Stereocontrolled Polyethers

**DOI:** 10.1002/cplu.202500540

**Published:** 2025-10-30

**Authors:** Teo Borst, Stefan Naumann

**Affiliations:** ^1^ Institute of Macromolecular Chemistry Albert‐Ludwigs‐University of Freiburg Stefan‐Meier‐Straße 31 79104 Freiburg Germany

**Keywords:** epoxides, organocatalysis, polyethers, polymerization catalysis, stereoselectivity

## Abstract

The stereoselective polymerization of racemic epoxides represents an increasingly powerful route to materials with tailored properties. Progress in this field is closely connected to advanced catalyst design and a growing understanding of polymerization mechanisms. This review briefly summarizes the historical development of the field and then focuses on research covering the past 10 years. Polyethers, already widely employed both for the mass market and for highly specialized applications, can be expected to gain further functionality and applicability based on these advances. A succinct final chapter provides an outlook, highlighting where stereocontrolled polyethers, in particular isotactic polymers, have already found fruitful application.

## Graphical abstract

This review summarizes the current state of stereoselective epoxide polymerization, focusing especially on the past 10 years. Polymerization mechanisms, catalyst design strategies (both organometallic and metal‐free), and applications of stereocontrolled polyethers are discussed, including their use as thermoplastic materials or regarding interfaces and self‐assembly.

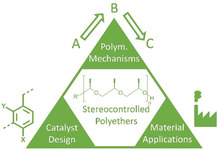

## 1 Introduction

Polyethers form an intriguing, versatile class of polymers that has found widespread application.^[^ ^1^ ^,^ ^2^ ^]^ This includes their usage as polyol components in polyurethane production,^[^ ^3^ ^]^ as lubricants,^[^ ^4^ ^]^ in cosmetics, skin care, and other daily use products,^[^ ^5^ ^]^ as well as in numerous medical applications (e.g., PEGylation)^[^ ^6^ ^,^ ^7^ ^]^ or for electrochemical devices (electrolyte).^[^ ^8^ ^,^ ^9^ ^]^ Polyether‐based surfactants are routinely employed and are crucial additives for many technical products, such as coatings, inks, or foams.^[^ ^10^ ^,^ ^11^ ^]^ This list could be easily extended for other examples.

Interestingly, this broad range of polyether products stems from few and simple building blocks, in particular ethylene oxide (EO) and propylene oxide (PO). While other cyclic ethers can also be used as feedstock, such as the five‐membered oxolane^[^ ^12^ ^]^ (tetrahydrofuran) or the four‐membered oxetane,^[^ ^13^ ^]^ the vast majority of commercially relevant products are based on epoxides. Both PO and EO can be polymerized via anionic polymerization using simple bases such as potassium hydroxide or potassium alkoxides. However, in the case of PO, more sophisticated catalyst systems are typically required to suppress side reactions and to achieve higher molecular weights. In this context, double metal cyanide (DMC) catalysts have become particularly well established and widely employed in both academic and industrial polyether synthesis.^[^ ^14^ ^]^ For a more comprehensive overview of polyether synthesis and applications, also beyond simple EO and PO, excellent literature can be recommended.^[^ ^1^ ^,^ ^2^ ^,^ ^15^, ^16^, ^17^ ^–^ ^18^ ^]^

Poly(ethylene oxide)/poly(ethylene glycol) (PEO/PEG) is a water‐soluble, semicrystalline material with low toxicity and high compatibility in strongly polar or ionic environments. Commercial PPO, poly(propylene oxide), on the other hand, is a lipophilic, fully amorphous compound with an oily appearance.^[^ ^1^ ^,^ ^2^ ^]^ While PEO and PPO thus already cover very different properties and are valuable on their own, the copolymerization of both monomers further extends the property profile of the thus accessible polyethers. Various polymer architectures (block copolymers, statistical copolymers, hyperbranched polyethers) can be targeted and many of those are used commercially.^[^ ^19^, ^20^, ^21^ ^–^ ^22^ ^]^ In sum, the broad applicability outlined above is the result of a significant compositional versatility, not even considering that other epoxides (such as glycidyl ethers^[^ ^23^ ^]^) also constitute promising alternatives.

With this background, it may be surprising that polymer tacticity, typically a key tuning parameter for polymers with chiral centers, has to date never played a relevant role in polyether products manufactured on a larger scale. This comes in spite of the fact that, for example, a sufficient degree of isotacticity provides PPO with the ability to crystallize, in stark contrast to its *atactic* congener (*at*‐PPO). Indeed, *isotactic* PPO (*it*‐PPO) displays a well‐defined melting point, up to 70 °C for a stereoerror‐free sample. Similar is found for other polyethers derived from various epoxides (see **Table** **1**).

**Table 1:** Examples for epoxide monomers and the respective melting temperatures of the corresponding *isotactic* polymers.^[^ ^48^ ^]^ 1,2‐butylene oxide (BO); 1,2‐hexylene oxide (HO); *n*‐butyl glycidyl ether (BGE); phenyl glycidyl ether (PhGE).

monomer	polyether	level of isotacticity [% *mm*]	*T*_m_ [°C]
PO	*it*‐PPO	>99	67
BO	*it*‐PBO	>99	22
HO	*it*‐PHO	>99	57
BGE	*it*‐PBGE	91	21
PhGE	*it*‐PPhGE	>99	193

Hence, stereocontrol as a tool has the potential to render some polyethers interesting bulk materials, for example, as engineering plastics for specific applications (see section 5), however, the number of suitable examples in literature is as yet relatively small. Likewise, it is clear that the interfacial properties of polyether‐based surfactants will be impacted by tacticity. Given the broad use of polyethers in just such setups (e.g., surfactants), the implications are significant. In sum, stereocontrolled aliphatic polyethers represent a field of research that provides exciting opportunities for further innovations and applications, in spite of the relatively small body of literature dealing with this subject to date.

In order to unlock polymer tacticity as a meaningful tool for improved polyether materials as outlined above, it is necessary to make use of the *racemic* monomer feedstock—polymerization of the enantiopure epoxide, of course, delivers *isotactic* polyether but is prohibitively expensive outside academic interest. Thus, stereoselective polymerization catalysts are required, ideally in a way that allows for their use as a “drop‐in” technology. This compatibility with existing processes also relates to functional group tolerance, a highly desired and immensely helpful feature for any catalyst. Thus, a functional group tolerant, stereoselective catalyst for the polymerization of epoxides can be expected to be less vulnerable to catalyst poisoning by impurities. Further, this could enable the polymerization of functionalized epoxides or the usage of functional (macro)initiators, extending the possibilities for stereocontrolled polyethers. Interestingly, such features are now increasingly possible (section 4).

The following considerations focus on catalyst design and understanding of polymerization mechanisms (sections 2‐4), followed by a brief discussion of current and prospective applications of stereocontrolled polyethers. In this context, the interested reader is also referred to an excellent review by Coates and coworkers, which covers the field until 2014.^[^ ^24^ ^]^ This work also discusses important underlying concepts, such as the determination of polyether tacticity via NMR, which will not be reiterated here. In contrast, the review presented here is mainly interested in newer developments over the past ten years.

## 2 Early Examples

**Scheme 1:**
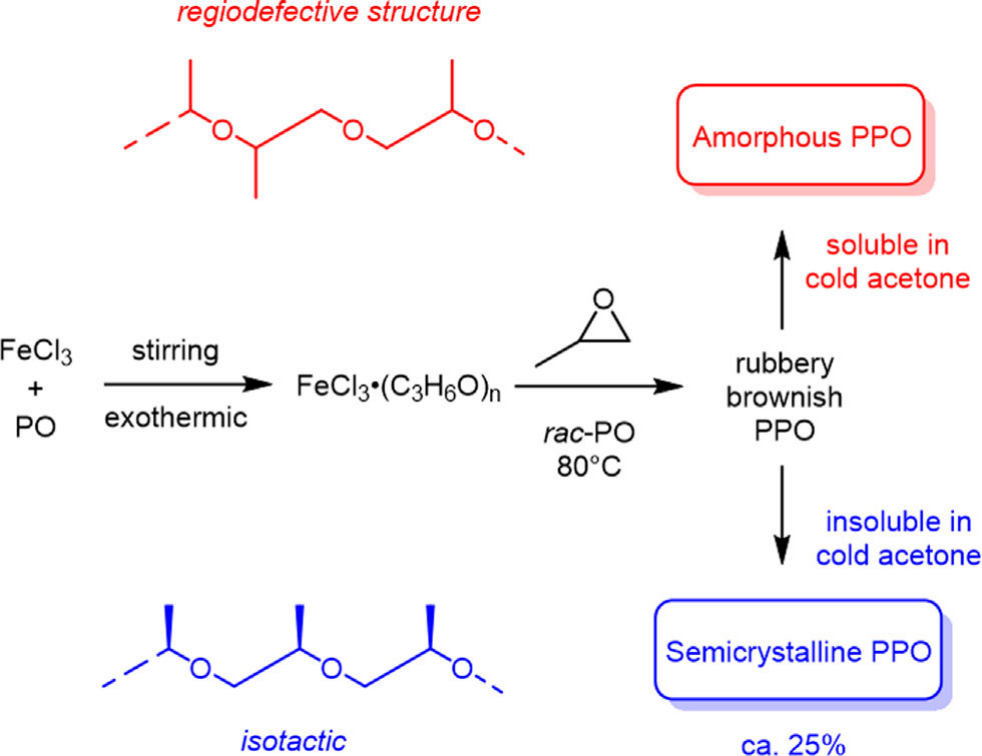
PPO as prepared via the Pruitt–Baggett‐catalyst.

In April 1955, a US patent by M. E. Pruitt and J. M. Baggett (of Dow Chemical Company) was published, whereby the polymerization of *racemic* PO (*rac*‐PO) was described to result in a solid resin.^[^ ^25^ ^]^ This was noted as exceptional, since the typical result of PO polymerization is *at*‐PPO, a fully amorphous substance of oily, liquid appearance. The authors achieved this by the addition of PO to anhydrous FeCl_3_ or FeBr_3_ under vigorous stirring, resulting in an exothermic reaction and the formation of a discolored “semisolid” residue of complex salts with the empirical formula of FeX_3_•(C_3_H_6_O)_*n*_. This residue was then used to polymerize PO, yielding the solid, rubbery resin‐like material mentioned above. Typical reaction conditions were *T* = 80 °C and relatively long reaction times (40–180 h). It was established that the product was a mixture of *it*‐PPO, the minor component, as a white, fluffy, semicrystalline polymer with a melting point of 70 °C, and amorphous PPO as the majority product (**Scheme** **1**).^[^ ^26^ ^]^ Interestingly, while the semicrystalline component was found to be a racemic mixture of all‐(*R*) and all‐(*S*) configured polyether chains, respectively, the amorphous material was found to display a high proportion of regiodefects (head‐to‐head vs. head‐to‐tail selectivity), explaining its inability to crystallize.^[^ ^27^ ^]^

Generating a large proportion of amorphous PPO is a major downside of the Pruitt–Baggett catalyst. Unfortunately, rational optimization of the catalyst is hampered by the absence of clear information on the active species and the polymerization mechanism. Most likely, iron alkoxides are formed in situ and form the active species, for which monomeric,^[^ ^28^ ^]^ dimeric,^[^ ^27^ ^]^ and trimeric^[^ ^29^ ^]^ structures have been proposed, among others.

The next wave of research activities was mainly directed at the investigation of (partially hydrolyzed) organometallic compounds, especially trialkyl aluminum and dialkyl zinc. Already in 1959, Price employed AlEt_3_ and Al(^*i*^Bu)_3_ to polymerize *rac*‐PO.^[^ ^30^ ^]^ It was assumed that Al alkoxides, similar to the Fe alkoxides most likely operative in the Pruitt–Baggett catalyst, could thus be formed in situ, potentially delivering *it*‐PPO. Results were rather disappointing, however, as reaction over 14 days delivered low yields (ca. 50%), and only 2–3% of the material was composed of the desired *isotactic* polyether. Barron later clarified some aspects, including the importance of complex formation between the formed alumoxane and PO.^[^ ^31^ ^]^ Using the latter complexes for polymerization resulted in a high proportion of *isotactic* diad placement (*m*).

**Scheme 2:**
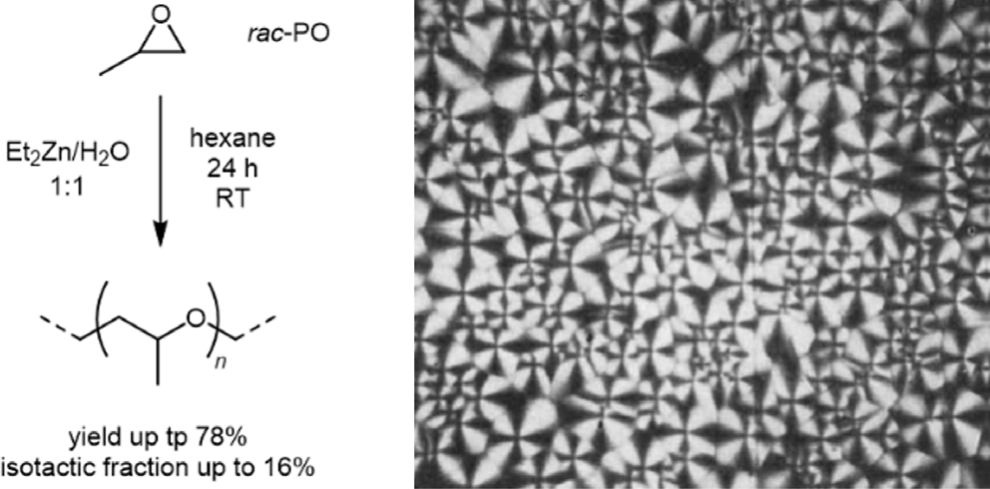
Left: Preparation of *it*‐PPO by the action of diethyl zinc as reported by Furukawa. Right: Film of the thus prepared polymer, displaying the spherulite structure typical for a semicrystalline polymer. Reproduced with permission.^[^ ^32^ ^]^ Copyright 1959, Wiley.

Also in 1959, Furukawa and coworkers used diethyl zinc (Et_2_Zn) together with cocatalysts (water, alcohols) in a certain ratio (e.g., 0.5–2.0 molar equivalents, relative to the zinc compound) to polymerize *rac*‐PO.^[^ ^32^ ^]^ The resulting PPO contained a fraction insoluble in cold acetone (up to 16% of the total PPO mass). This fraction was shown to be highly *isotactic*, forming films with well‐developed spherulites (**Scheme** **2**). Hurst later showed that sterically hindered epoxides, *tert*‐butyl ethylene oxide (^*t*^BEO) and styrene oxide (SO), can also be successfully polymerized via the ZnEt_2_/water route, yielding semicrystalline material in both cases.^[^ ^33^ ^]^

**Figure 1:**
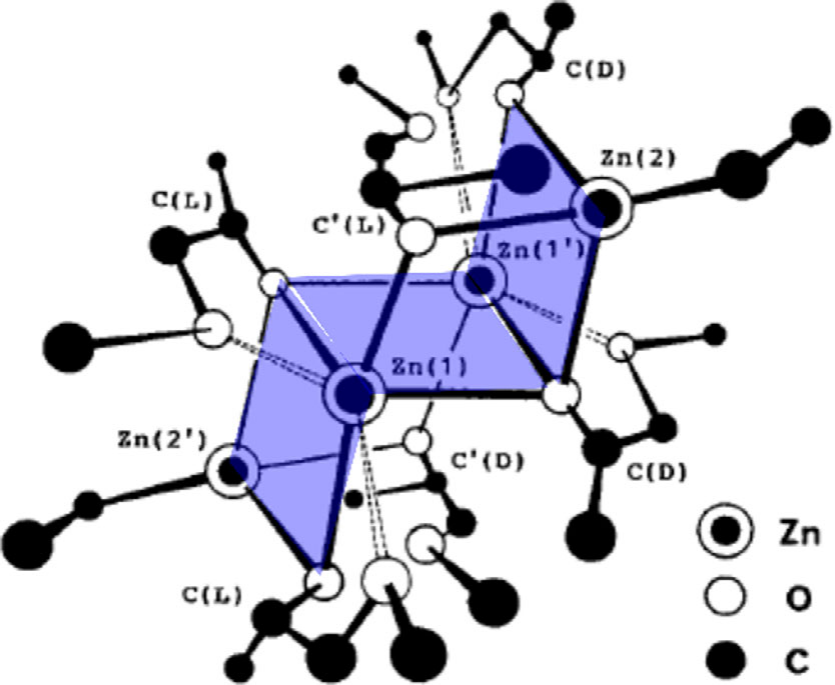
Catalyst **1**, as derived by Tsuruta and coworkers. Modified to emphasize the “chair” arrangement. Reproduced with permission.^[^ ^34^ ^]^ Copyright 1988, Wiley.

Tsuruta and coworkers expanded on the work conducted with Et_2_Zn by studying a range of zinc clusters prepared via the reaction of diethyl zinc with alkoxyalcohols.^[^ ^34^, ^35^ ^–^ ^36^ ^]^ The resulting compounds were characterized, including via single crystal X‐ray analysis, and employed for the polymerization of epoxides, mainly PO. This work was significant for a number of reasons. First, the choice of the alkoxyalcohol was decisive for the stereoselectivity of the resulting catalyst. The most successful example from the studied series was obtained by application of *racemic* 1‐methoxy‐2‐propanol. The resulting cluster structure (**1**, **Figure** **1**) was described by the authors as “chair‐like”. Application of 2‐methoxyethanol or methoxide, in contrast, delivered inferior results. Second, ^13^C NMR analysis was employed to reveal the diad/triad stereosequences in the polyether chains, moving away from older and imprecise techniques reliant on solvent fractionation (labeling PPO soluble in cold acetone (amorphous/*atactic*) or not (semicrystalline/*isotactic*)). Thus, it was found that **1** delivered PPO with *isotactic* diad (*m*) and triad (*mm*) sequences of 81% and 75%, respectively (*T* = 35 °C). In fact, by considering the relative abundance of *mm*, *mr* (heterotactic) and *rr* (syndiotactic) triads, the authors could substantiate that propagation statistics characteristic for enantiomorphic catalyst site control apply—hence stereocontrol is exerted by the chirality of the catalyst coordination site.^[^ ^34^ ^]^ With *m* = 81%, the PPO resulting from the action of **1** can therefore be considered strongly *isotactic* enriched (*m* = 50% signifies atactic material), yet the isotacticity level was still not high enough to render the material able to crystallize. Similar to the above approaches, Tsuruta and coworkers investigated diethyl magnesium (Et_2_Mg) for the purpose of polymerizing *rac*‐PO.^[^ ^37^ ^]^ It was found that it makes a difference whether the dialkyl species or the dialkoxide formed in situ is considered, with the latter showing no stereoselectivity at all. Overall, the effects were small; more recently, Mejia has revisited the idea of using Grignard‐type precatalysts for the generation of *it*‐PPO (see section 4).

In a related way, yet with the additional presence of a ligand, Vandenberg and coworkers already in the 1960s published work on a catalyst resulting from aluminum trialkyl, water, and acetylacetonate.^[^ ^38^, ^39^ ^–^ ^40^ ^]^ While the structure of the formed and active species was not precisely determined at the time and later investigated by Lynd and coworkers (see section 4), this catalyst nonetheless proved highly active, yielding, for example, unusually high molar masses of PPO, copolymers with other epoxides and polyether‐based elastomers.^[^ ^41^ ^,^ ^42^ ^]^ The Vandenberg catalyst also prove to be especially valuable for the polymerization of epichlorohydrin, as pointed out by Ferrier and coworkers.^[^ ^16^ ^]^ Polymerization of substituted epoxides typically yields moderately *isotactic*‐enriched polyethers when the Vandenberg catalyst is employed.

**Figure 2:**
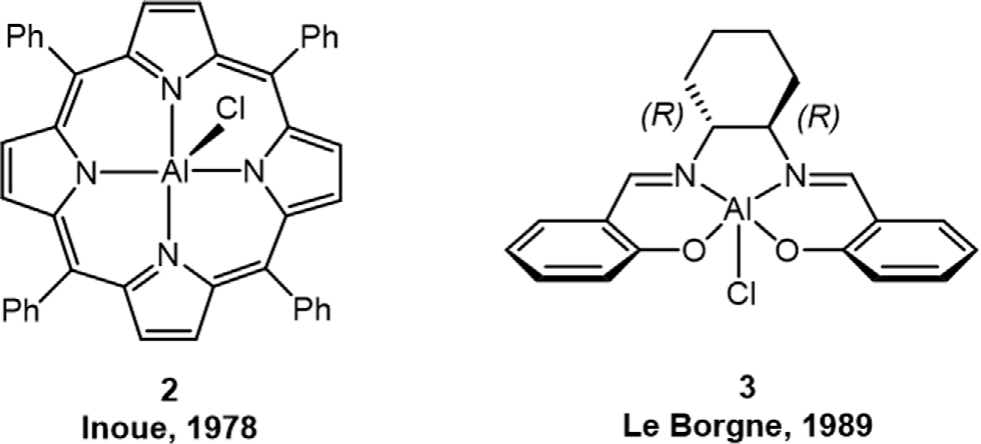
Early examples for well‐defined catalyst complexes with the ability to deliver *isotactic*‐enriched PPO.

Following research efforts were directed at the preparation of well‐defined, single‐site complexes. In 1978, Inoue published catalyst **2**, whereby a rigid porphyrin‐derived ligand coordinates to an Al(III) metal center (**Figure** **2**).^[^ ^43^ ^]^ In a study focusing on epoxide/CO_2_ copolymerization, it was found that **2** delivered PPO with *m* = 69% (20 °C, *M*_*n*_ = 8100 g mol^−1^, *Ð*_M_ = 1.10). A chain‐end control mechanism without penultimate effect was proposed; hence, the stereocenter of the last PO monomer added to the growing polymer chain influences stereoselectivity with a certain degree of isoselectivity.

Also working on aluminum complexes, Le Borgne and coworkers employed the well‐known “salcy” ligand scaffold (*N*,*N*’‐bis(2‐hydroxybenzylidene)−1,2‐cyclohexanediamine) to synthesize complex **3** (Figure 2).^[^ ^44^ ^]^ This catalyst (*R*,*R*‐configuration) was then applied for the generation of oligomeric PO. Indeed, a moderate preference for consuming the (*S*)‐PO over enchaining the (*R*)‐PO was observed (k_(*S*)_/k_(*R*)_ = 1.28, *T* = 60 °C). After more than 100 h of polymerization time and a conversion of 80% this corresponded to an enantiomeric excess (*ee)* of 18.5% in the remaining monomer. Chlorine content analysis suggested that initiation proceeds via insertion in the Al—Cl bond; propagation then ensues via the thus formed Al—O bond. Preferential coordination of the (*S*)‐PO to the Al(III) center due to steric effects by the chiral backbone was suggested to explain the observed selectivity.

## 3 The Coates Catalysts

The above is not a complete reiteration of the early research efforts directed at understanding and improving the ability to produce *isotactic* polyethers; an encompassing review can be found elsewhere.^[^ ^24^ ^]^ However, it is interesting to note that the field moved from using (partially) hydrolyzed alkyl metal compounds, often resulting in compositions of uncertain chemical structure, to well‐defined, homogeneous catalysts. Still, up to this point, stereoselective epoxide polymerization suffered from either low selectivity or the presence of amorphous by‐products (often in large proportion relative to the desired semicrystalline product), which had to be removed by solvent extraction.

This situation only changed in the 2000s, when the research group of Coates identified a family of homobimetallic catalysts that were not only found to be highly selective but also allowed for rational optimization of the catalyst structure. This group of compounds is here referred to as Coates Catalysts and their development is briefly summarized in the following.

**Figure 3:**
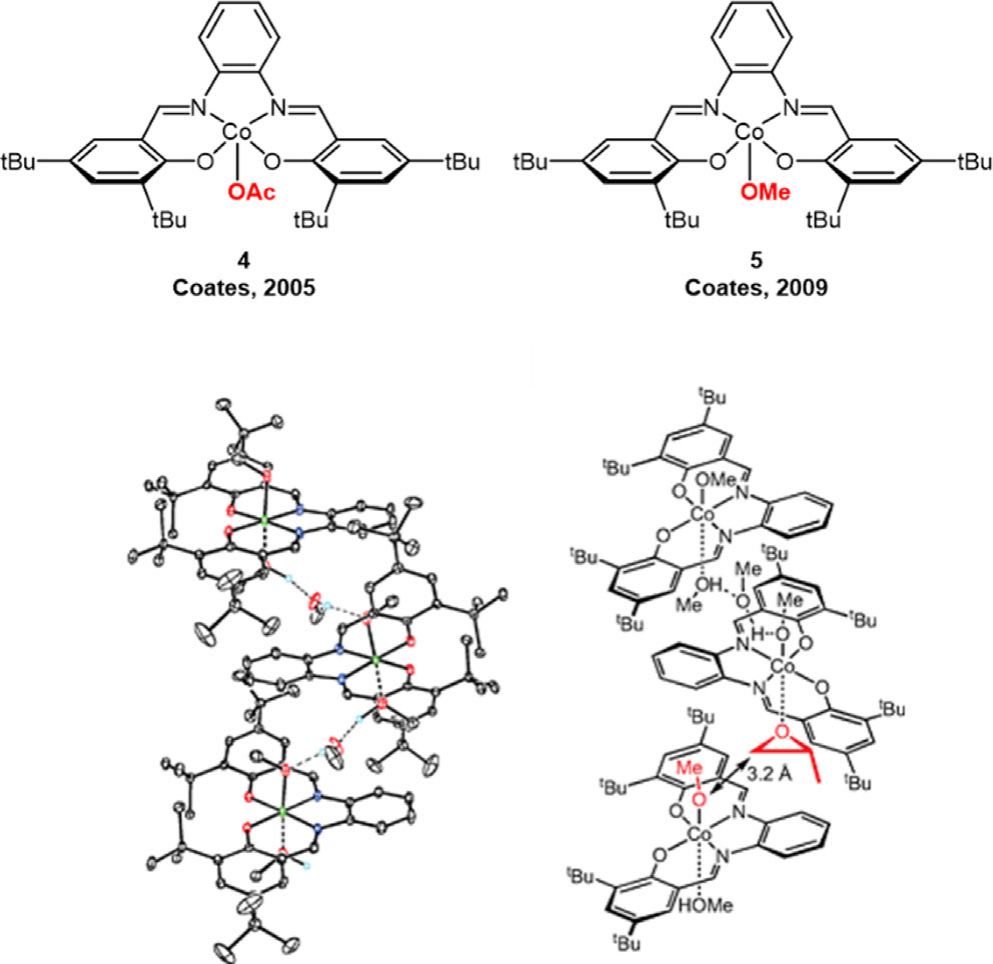
Top: Monometallic Co(III)‐salcy complexes for the generation of *it*‐PPO. Bottom: ORTEP‐drawing and chemical structure of the coordination polymer formed by **5**, with proposed polymerization mechanism. Reproduced with permission.^[^ ^46^ ^]^ Copyright 2009, Royal Society of Chemistry.

This step change in stereoselective epoxide polymerization started in 2005, when the group reported the achiral catalyst **4** (**Figure** **3**) to result in highly *isotactic* PPO (>99% *mm* triad placement) from *rac*‐PO feedstock.^[^ ^45^ ^]^ Indeed, the stereoselectivity was pronounced to such a degree that no side product and no NMR‐discernible stereoerrors could be observed. The latter ironically meant that the type of stereocontrol, chain‐end or catalyst‐site related, could not be determined from stereo‐error analysis. High molar masses (*M*_*n*_ = 250 000 g mol^−1^) and relatively broad molar mass distributions were observed (*Ð*_M_ = 1.5–2.5), alongside specific solvent effects and a selectivity optimum at 0 °C (at lower or higher temperature, both conversion and turnover frequency drop, while the level of stereoselectivity remains undisturbed up to 40 °C).

Considering Co(III) catalyst **4**, which carries both an acetate and salcy ligand (salcy = *N*,*N*’‐bis(3,5‐di‐tertbutylsalicylidine‐1,2‐benzenediamine), the origin of its remarkably enhanced performance is not immediately evident. This was elucidated in careful investigations, hampered by the hard‐to‐crystallize and paramagnetic **4**, where it was revealed that a heterogeneous polymerization is operative (only the nondissolved proportion of the catalyst is engaged in stereoselective polymerization).^[^ ^46^ ^]^ A substitution of the acetate for a methoxy ligand (**5**) finally enabled single‐crystal X‐ray structure analysis, proving that the catalyst arranges in a coordination copolymer (via the OMe ligands, Figure 3) with pseudo‐*C*_2_‐symmetry. This provides the chiral environment, in which the Co—Co distance is fixed at 713 pm, which in turn favors a bimetallic propagation step involving two moieties of **5**. Thereby, one Co(III) carries the initiator/propagating chain end while the neighboring Co(III) coordinates (and thus activates) the epoxide.

**Figure 4:**
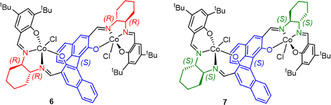
Bimetallic Co(III)‐Co(III) polymerization catalysts as reported by Coates and coworkers.

The above insight was successfully transferred to a fully homogeneous, soluble analog (**6**, **Figure** **4**), containing the above‐mentioned bimetallic core.^[^ ^47^ ^]^ This complex contains a binaphthyl backbone, imparting a decisive axial chirality, to which the cobalt centers are attached via salicylidene motifs. The Co—Co distance is in a range of 500–700 pm (the binaphthyl backbone permits some dihedral flexibility). **6** requires activation with an ionic cocatalyst (bis(triphenylphosphine)iminium acetate, [PPN][OAc]) to enable conversion of epoxides, yet such polymerizations display excellent selectivity. Moreover, a broad range of monomers (PO: 99% *mm*, BO: 99% *mm*, HO: 99% *mm*, styrene oxide (SO): 94% *mm*) is suitable.^[^ ^47^ ^,^ ^48^ ^]^ The chirality of the binaphthyl linker (marked blue, Figure 4) determines the preference for a given enantiomer of the epoxide;^[^ ^49^ ^]^ in contrast, the stereocenters located on the cyclohexyldiamine building block (red/green) are less impactful—diastereomers **6** and **7** are selective for the same epoxide enantiomer.^[^ ^24^ ^]^

For quantification of the stereoselectivities of these different complexes, a selectivity factor (*s*) can be established, defined as the ratio of k_*S*_/k_*R*_, the individual polymerization rates of, e.g., (*S*)‐PO and (*R*)‐PO. The necessary data can be approximated via polymerization of the enantiopure monomers or determined by analyzing the residual monomer using gas chromatography‐mass spectrometry equipped with a suitable chiral column.^[^ ^47^ ^]^ For a highly selective setup such as the one based on **6** (*s*_(PO)_ = 370, *s*_(BO)_ = 330, *s*_(HO)_ = 260, all at *T* = 0 °C) this means that monomer conversion practically stops once the preferred enantiomer of the epoxide has been consumed. This is beneficial for procuring enantiopure, residual monomers, yet if all of the epoxide feedstock is to be used for making polymer, *racemic* catalyst mixtures have to be employed. Indeed, *rac*‐**6** can achieve this, still delivering excellent selectivity, yet with the significant downside that both catalyst enantiomers have to be made separately, because inseparable diastereomer mixtures form if a one‐pot synthesis is attempted. This issue can be circumvented by application of an achiral diamine building block (ethylene diamine instead of cyclohexyldiamine).^[^ ^50^ ^]^ By suitable tuning of the reaction parameters and optimization of the required ionic cocatalyst, still a relatively high selectivity of >97% *mm* was achieved, alongside fast reaction and high molar masses (>100 000 g mol^−1^). Polydispersity was in the range of 1.4–1.8 and a range of epoxide monomers could be polymerized, including electron‐poor feedstock (e.g., epoxides substituted with Ph‐ or CF_3_‐groups).

## 4 Recent Advances

Progress in the field of stereoselective epoxide polymerization, as outlined above, was achieved by moving from rather ill‐defined catalysts or catalyst mixtures to well‐defined organometallic complexes, culminating in the powerful bimetallic polymerization catalysts by Coates, described in the previous section. While impressive, these advances have not led to the commercialization of *isotactic* or otherwise stereo‐controlled polyethers. In part, this can be attributed to some significant downsides that even the most advanced catalysts at the time displayed. Within the past ten years, research efforts have been directed at finding solutions to these downsides. Major aspects of interest encompassed, for example, the utilization of chain transfer agents (CTAs), the ability to obtain narrower molar mass distributions, the broadening of functional group tolerance or the simplification of the synthetic accessibility of catalysts—all important factors which render stereoselective epoxide polymerization more practicable and versatile. Remarkable advances have been achieved, and the respective strategies will be discussed in the following. First, classic organometallic approaches will be considered, followed by a subsection on organocatalysis.

### 4.1 Metal‐Based Polymerization Catalysts

Efficient use of all the epoxide feedstock (both enantiomers) and improved molar mass control of the resulting polyether had remained important aims not readily achieved by the Co(III)‐based bimetallic Coates Catalysts. Relatively slow initiation and rapid polymerization entailed broadened molar mass distributions and complicated the tailoring of the overall *M* *_n_*. At the same time, using both epoxide enantiomers with high selectivity means using *racemic* catalyst mixtures (with each catalyst enantiomer preferring a specific epoxide monomer enantiomer). Consequently, controlling molar masses via the employment of alcohol‐type CTAs then seems like a promising choice. However, the exchange, or chain shuttling, of growing polyether chains between catalysts (potentially of opposite stereoselectivity) introduces an additional layer of complexity to the reaction. The outcome depends strongly on the relative rates of propagation and chain shuttling. If the propagation rate is lower than or comparable to the shuttling rate, the frequent switching between catalysts will scramble any stereocontrol, leading to *atactic* polymers. In contrast, if the rate of chain shuttling is lower relative to propagation, the formation of stereoblock polyethers is favored.

**Scheme 3:**
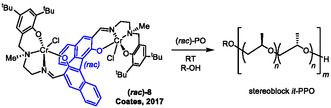
Chain‐shuttling polymerization of PO employing a bimetallic Cr(III) catalyst. Cocatalyst (not shown): bis(triphenylphosphine)iminium pivalate, [PPN]OPiv.

These aspects were investigated by Coates and coworkers in a study published in 2017.^[^ ^51^ ^]^ Thereby, a homobimetallic Cr(III) catalyst was employed as a readily accessible *racemic* mixture (**Scheme** **3**). The ligand system is chiral, again induced by a binaphthyl motif (**8**), and can be constructed in a one‐pot synthesis. Characterization of these chromium catalysts proved to be somewhat difficult as NMR investigations are hampered by the paramagnetic Cr(III), and crystallization of **8** did not succeed.

Nonetheless, application of (*S*)‐**8** showed the compound to be selective for (*S*)‐PO with *s* = 60, rendering it a suitable candidate for studying room temperature polymerizations with (*rac*)‐**8** in the presence of 1,6‐hexanediol as CTA and *rac*‐PO as monomer. Interestingly, it was found that indeed the molar mass could be regulated by adjusting the ratio of PO to CTA. Furthermore, detailed analysis of the stereoerrors present in the formed polymer revealed a ratio of (*mr*) = (*rm*) > (*rr*), which is diagnostic for the formation of stereoblock *it*‐PPO (Scheme 3).^[^ ^52^ ^,^ ^53^ ^]^ High isotacticity was maintained (*mm* > 87%).

Fittingly, when *T* was raised to 120 °C, the formed polyether was either still highly *isotactic* (no CTA) or predominantly *atactic* (CTA present). This is in line with the assumption that higher temperatures will favor rapid exchange of growing polyether chains when CTA is present. In sum, this entails the possibility of a) controlling molar masses in a convenient way, enabling also *α*,*ω*‐dihydroxylated polyether products, b) full conversion of the epoxide feedstock (both enantiomers), and c) still very high isotacticity. Prerequisites are fitting kinetics (of propagation vs. chain transfer) and readily accessible *racemic* catalyst structures.

**Scheme 4:**
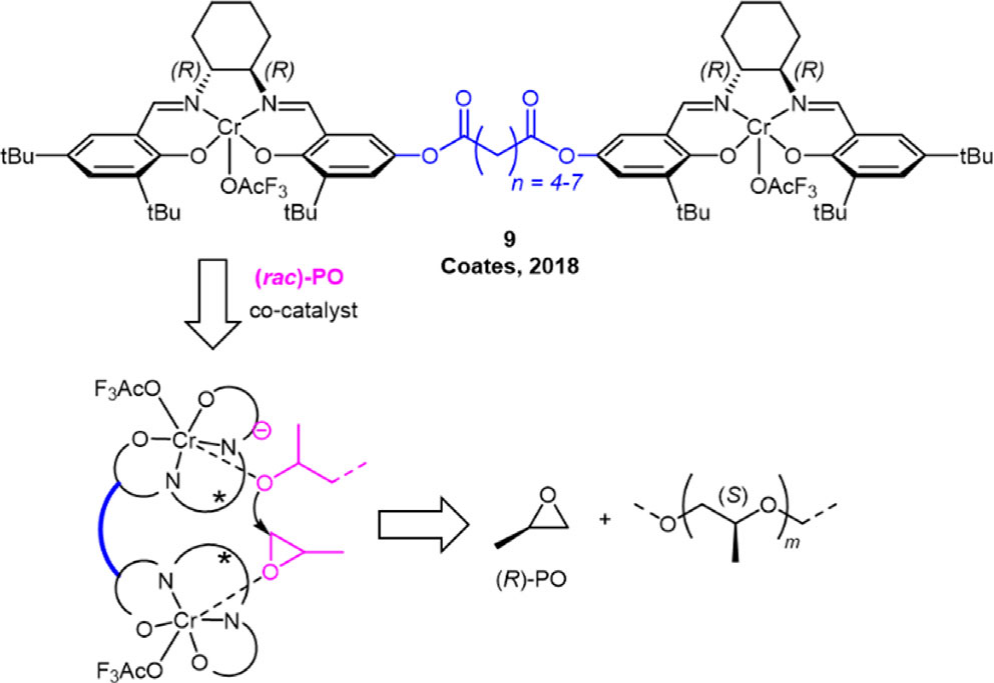
Bimetallic chromium catalysts with flexible linker motif for the stereoselective polymerization of PO. Cocatalyst = [PPN]Cl.

In 2018, the same group investigated strategies to further improve on the obtained molar mass distributions.^[^ ^54^ ^]^ By using a motif previously investigated for related applications by Jacobsen and Chen,^[^ ^55^ ^,^ ^56^ ^]^ connecting two chromium salen species via a flexible linker, this aspect could be further improved (**Scheme** **4**). Linker length and initiating group were found to be crucial parameters to optimize results. Using catalyst series **9** with *n* = 4–7, a trifluoroacetate ligand and hexanediol as CTA at room temperature, *mm* > 87% was achieved in the polymerization of *rac*‐PO, alongside narrow molar mass distributions (*Ð*_M_ < 1.10) and controllable molar masses of up to *M*_*n*_ > 50 kg mol^−1^, highlighting a significant advance compared to previous generations of Coates‐type catalysts. The much narrower polydispersity of the resulting PPO was attributed to a combination of slower polymerization (relative to initiation) and the flexible character of the catalyst. To achieve the bimetallic propagation step, the catalyst has to “backfold” to assemble in a suitable conformation and Cr—Cr distance to enable monomer enchainment. Accordingly, *n* has a very relevant impact on performance, whereby for *n* = 4, 5, 6, and 7 under identical condition conversions of 0%, 35%, 46%, and 12% were observed. Notably, however, no substituted epoxides other than PO could be successfully polymerized by this catalyst type.

**Scheme 5:**
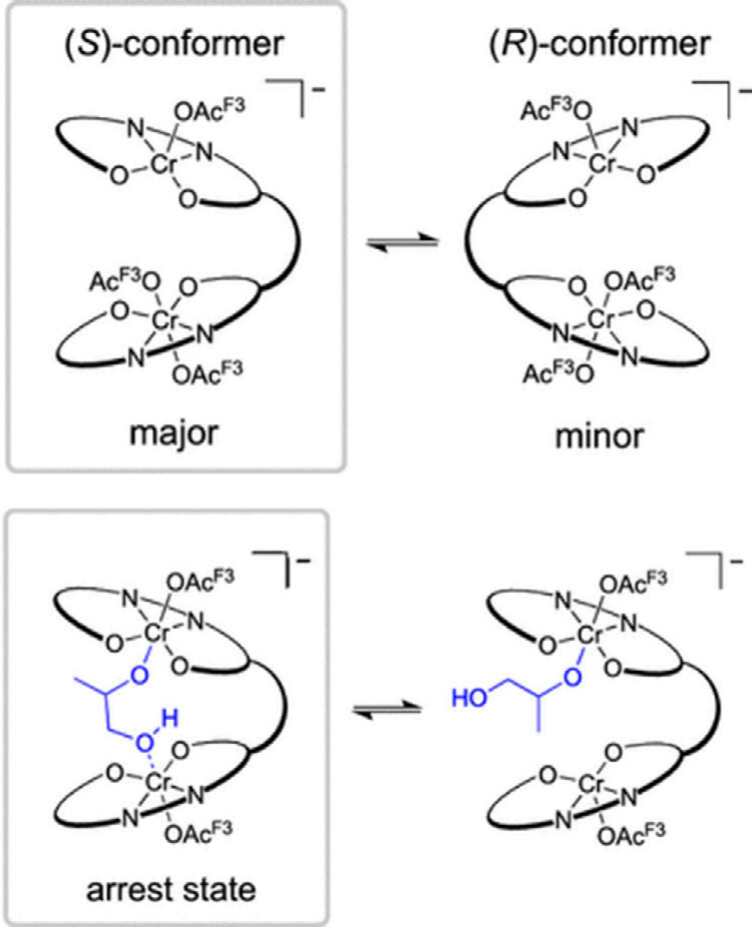
Top: Proposed complex formation of **9** (*n* = 6). Bottom: 1,2‐hydroxypopanolate‐mediated resting state, coresponsible for the observed induction time, and equilibrium with the active species (= species with free coordination site for monomer). Reproduced with permission.^[^ ^57^ ^]^ Copyright 2020, American Chemical Society.

In a joint effort, the groups of Zare, Zimmermann, Waymouth, and Coates further illuminated the mode of action of catalyst **9** (*n* = 6) by a combination of electrospray ionization‐mass spectrometry experiments and density functional theory (DFT) calculations.^[^ ^57^ ^]^ For one, DFT suggested that this catalyst folds to predominantly form the (*S*)‐conformer (**Scheme** **5**, top) during propagation. This behavior seems to be governed by the chirality of the cyclohexanediamine backbone. Moreover, it was found that resting states resulting from the presence of water entail the substantial induction times that have been observed for **9**. One arrest state is a µ‐hydroxide complex, while the more severe slow‐down originates from a bridged 1,2‐hydroxypropanolate complex (Scheme 5, bottom), which can form in the presence of PO and water. Importantly, this detailed understanding allowed for a description of reaction conditions to suppress the occurrence of induction times, namely rigorous drying of all polymerization components as well as the avoidance of any diol‐type initiators with vicinal hydroxy groups.

Interestingly, catalyst **9** (*n* = 6) was also successfully employed to generate (statistical) polyether‐polyester copolymers.^[^ ^58^ ^]^ The presence of ester bonds in the polymer chains installs a type of degradability (hydrolysis) that is not inherent to polyether homopolymers (under certain conditions, also pure polyethers degrade, see section 5). At the same time, the *isotactic* polyether segments can be expected to increase the mechanical properties of the resulting material.

**Scheme 6:**
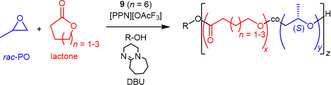
Copolymerization of PO and lactones using a chain shuttling approach between bimetallic chromium catalyst **9** and organocatalyst DBU.

With this motivation, the copolymerization of PO with several lactones (*γ*‐butyrolactone, GBL, *δ*‐valerolactone, VL, *ε*‐caprolactone, CL) and lactide was investigated. To enchain both types of monomers, a chain‐shuttling approach was employed (**Scheme** **6**), whereby polymerization of the epoxide occurred at the chromium sites while lactone ring‐opening was effected by a guanidine‐type organobase (1,8‐diazabicyclo[5.4.0]undec‐7‐ene, DBU). By regulating the feed ratio, materials closely resembling *it*‐PPO (low ester content) or amorphous copolymers (high ester content) were accessible. The *it*‐polyether segment length depended on the proportion of CTA present, in accordance with the chain‐shuttling concept. In general, copolymerization of VL worked best, while also CL and GBL could be incorporated. Using lactide, no copolymers were generated.

While continuing research efforts are invested in a further improvement of Coates‐type catalysts, also other stereoselective polymerization systems have found increasing attention in recent years. In 2018, Lynd, Mendoza‐Cortes, and coworkers^[^ ^59^ ^]^ revisited the Vandenberg catalyst first described in the 1960s (see section 2). While the Vandenberg catalyst continues to be important today and has been successfully employed for the polymerization of various epoxides and other monomers,^[^ ^16^ ^,^ ^60^ ^,^ ^61^ ^]^ the structure and polymerization mechanism have remained unclear. Vandenberg had combined acetylacetone (1 eq.), water (1 eq.), and trialkylaluminium (2 eq.), initially planning to block some of the coordination sites of the Al‐species formed in situ for a better mechanistic understanding. Unexpectedly, rather than tuning down reactivity, this led to a powerful, highly active polymerization system.

**Figure 5:**
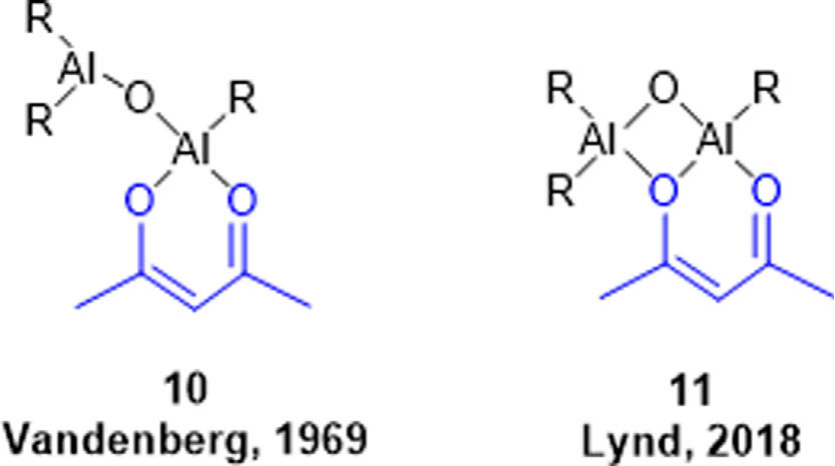
Structural proposals for the Vandenberg catalyst. R = Me, Et. Blue = acetylacetonate ligand.

From the stoichiometry of the catalyst synthesis and observation of the released gaseous byproducts, Vandenberg had originally proposed structure **10** (**Figure** **5**), a mono(µ‐oxo)‐dialuminium species.^[^ ^40^ ^]^ However, this structure cannot explain the moderate levels of isotacticity found in the resulting polyethers. In contrast, Lynd, Mendoza‐Cortes, and coworkers proposed a bis(µ‐oxo)‐dialuminium structure (**11**) as a key feature of the Vandenberg catalyst.^[^ ^59^ ^]^ The latter is a rigid structure, which was considered to enable a degree of isoselectivity, while **10**, in contrast, displays rotational freedom.

**Scheme 7:**
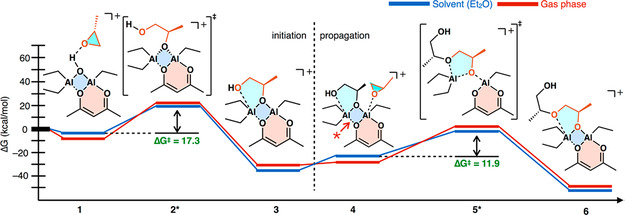
Reaction coordinate for the polymerization of PO via the Vandenberg catalyst as proposed by Lynd and coworkers. Reproduced with permission.^[^ ^59^ ^]^ Copyright 2018, American Chemical Society.

A combined DFT and experimental study revealed further intriguing details about the polymerization mechanism. Initiation is suggested to proceed from trace µ‐hydroxide species (**Scheme** **7**). Concerted ring‐opening then occurs via a transition state with a relatively low barrier (Δ*G* ^‡^ = 17.3 kcal mol^−1^, at 298 K for PO) to result in a structure where the ring‐opened monomer is bound at two points (both oxygen atoms) to the catalyst. For the propagating chain end, this corresponds to the ultimate and penultimate oxygen atoms being coordinated, forming a fixed configuration which imparts chirality to the propagating center. Indeed, a penalty of 2 kcal mol^−1^ was found when oppositely configured PO enantiomers are incorporated in the polyether chain, likely explaining the moderate isoselectivity observed for the Vandenberg catalyst. It should be noted that the mechanism involves a mono(µ‐oxo)‐dialuminium transition state (5*, Scheme 7) that settles back into the rigid bis(µ‐oxo)‐dialuminium structure during propagation. Thus, the acetylacetonate‐chelated Al species coordinates the monomer while the second Al center fixates the configuration of the polymer chain end by coordinating both the ultimate and penultimate oxygen atom. Based on this insight, a model compound was synthesized, which displayed similar levels of isotacticity as observed for the conventional Vandenberg catalyst.^[^ ^59^ ^]^ In spite of these significant advances, the authors acknowledged that the situation for the original catalyst may be more complex, since there is a mixture of different species that must be assumed (some of which produce the very high molar masses characteristic of the application of the Vandenberg catalyst).

It is very interesting to note that Lynd and coworker also employed the Vandenberg catalyst for the statistical copolymerization of epoxides and lactones/lactide.^[^ ^62^ ^]^ The investigated monomers encompassed EO, PO, BO, ECH, *rac*‐lactide and CL. Overall, seven different heterocopolymers were targeted and NMR signals, as well as gel permeation chromatography and optical behavior, supported statistical incorporation. For some of these, the copolymerization parameters were determined, revealing a gradient structure with a preference for the epoxide (e.g., *r*_PO_ = 2.81, *r*_LA_ = 0.36). While the tacticity of the polyether segments was not discussed regarding its potential impact on the properties of the resulting poly(ester‐ether) materials (maybe also in view of the rather moderate isoselectivity of the Vandenberg catalyst), it is clear that such polymer architectures and the required functional group tolerance will be a decisive feature for prospective applications of tacticity‐controlled polyether‐based materials (see section 5).

In 2017, Mejia and coworkers again looked at the application of magnesium alkyl compounds and their potential for stereoselective epoxide polymerization.^[^ ^63^ ^]^ While early investigations (see section 2) had delivered low selectivity under similar conditions,^[^ ^37^ ^]^ this group found ^*n*^Bu_2_Mg to be highly effective for generating well‐defined *it*‐PPO with *mm* > 99%, alongside controllable molecular weights of up to M_*n*_ > 50 kg mol^−1^ even at 40 °C. However, the underlying mechanism remains unclear; proposed catalyst structures were investigated via DFT and did not entail plausible stereoselection. More research regarding the application of Grignard‐type catalysts seems both necessary and rewarding, especially in view of the simplicity of such prospective catalysts.

### 4.2 Metal‐Free Polymerization Catalysts

All of the literature discussed in the previous sections employed some sort of metal‐based catalysts for the stereoselective polymerization of epoxides. Indeed, also when reviewing the field in a thoroughly encompassing manner,^[^ ^24^ ^]^ it is clear that metal‐free (organocatalytic) strategies have been completely absent from the literature since the earliest days of research on this topic.

However, in 2022, Naumann and coworkers revealed the first example for organocatalytic stereoselective polymerization of epoxides, resulting in *isotactic*‐enriched PPO, PBO, and PAGE, respectively.^[^ ^64^ ^]^ This was achieved by using two alkyl borane functionalities connected via a chiral linker.

Borane‐mediated (co)polymerization^[^ ^65^, ^66^ ^–^ ^67^ ^]^ received a surge in interest after Feng, Gnanou, and Hadjichristidis published their seminal work on epoxide/CO_2_ copolymerization, using triethylborane (Et_3_B) as a cocatalyst.^[^ ^68^ ^]^ Soon thereafter, it was demonstrated that this simple and commercially available molecule can bring about the highly controlled homopolymerization of epoxides, resulting in excellent control over molar masses and their distribution.^[^ ^69^ ^,^ ^70^ ^]^ Typical reaction conditions included CTA (such as BnOH) and an organobase as cocatalyst at *T* = 0 °C. Molar masses with *M*_n_ > 100 kg mol^−1^ and *Ð*_M_ < 1.10 could be readily obtained.

**Scheme 8:**
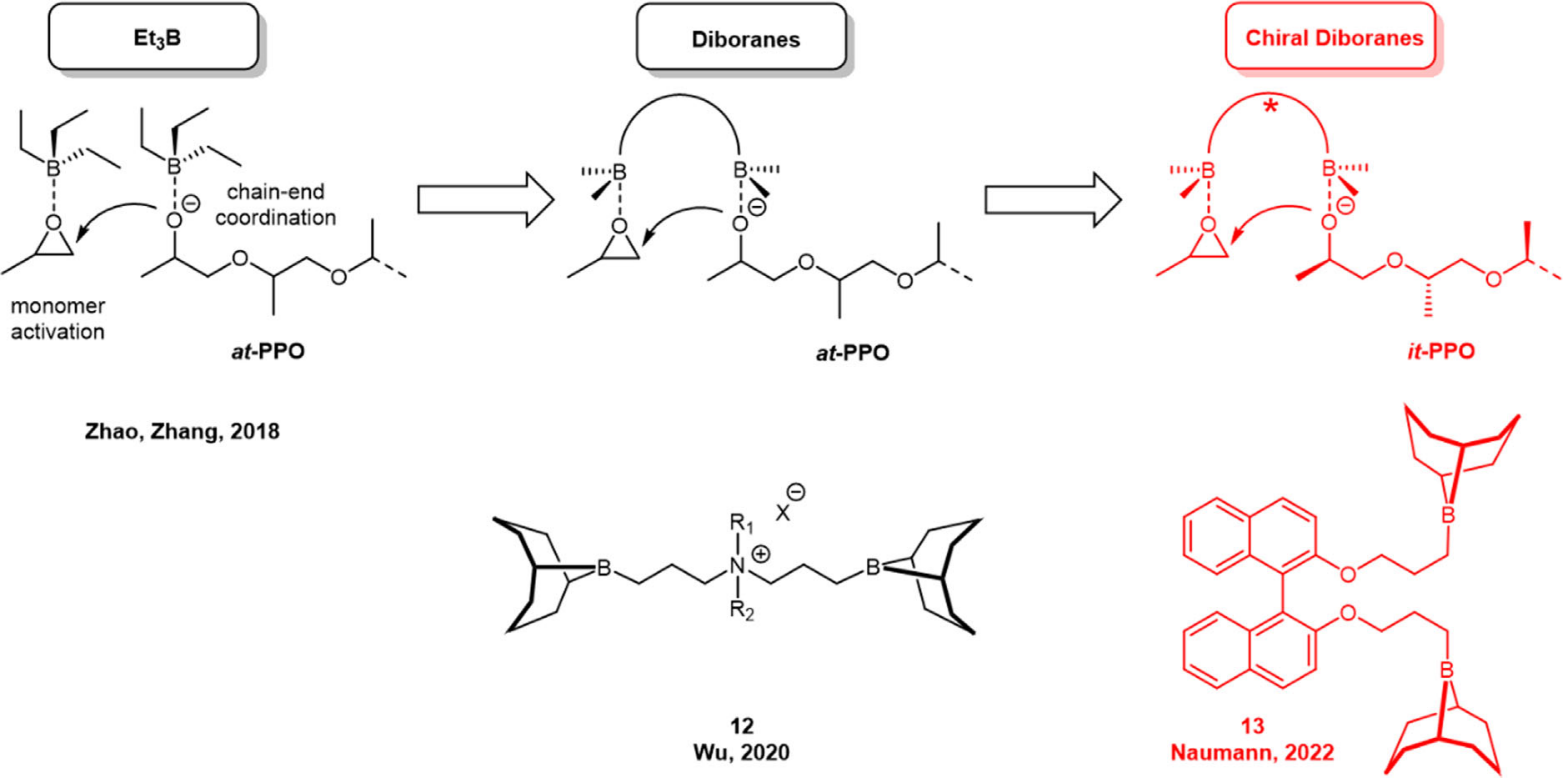
Development from monoborane to diborane to chiral diborane catalysts. The latter can be employed to prepare *isotactic*‐enriched polyethers.

Crucially, the polymerization mechanism was plausibly explained by invoking a dual role for the moderately Lewis‐acidic Et_3_B (**Scheme** **8**). Thus, the propagating chain end, deprotonated by the organobase cocatalyst, coordinates to the borane. This tunes down the reactivity of the oxyanionic species, significantly reducing its ability to undergo undesired side reactions such as transfer to monomer and likewise increases its tolerance for functional groups. Indeed, the propagating chain end is deactivated to such a degree that it can only react with activated (= borane‐coordinated) epoxide. In sum, this leads to a highly controlled yet still rapid monomer enchainment, following zero order kinetics with respect to the monomer. The functional group tolerance has been convincingly demonstrated in several studies, which reported successful polymerizations of monomers bearing functional groups that would typically undergo degradation under conventional polymerization conditions.^[^ ^71^, ^72^ ^–^ ^73^ ^]^

Considering the mechanism of the propagation step (Scheme 8), where two molecules of borane are required, it is clear that the polymerization will be susceptible to dilution. Indeed, the technically relevant presence of excess CTA (such as in a large‐scale reactor) could result in a very pronounced slowdown of the polymerization kinetics.^[^ ^69^ ^]^ To overcome this hurdle, Wu and coworkers developed so‐called diboranes, whereby the two boranes are linked together via an ammonium‐type bridge (such as **12**, Scheme 8). This structure ensures that the activated monomer is found in close proximity to the propagating chain‐end, enabling excellent rates of conversion even under dilution or in the presence of functionalities that compete for coordination to the borane (such as CTA). This led to a massively increased performance and outstanding results regarding epoxide homopolymerization and copolymerization, albeit of course still delivering *atactic* polyether.^[^ ^65^, ^66^ ^–^ ^67^ ^,^ ^74^, ^75^, ^76^ ^–^ ^77^ ^]^

**Scheme 9:**
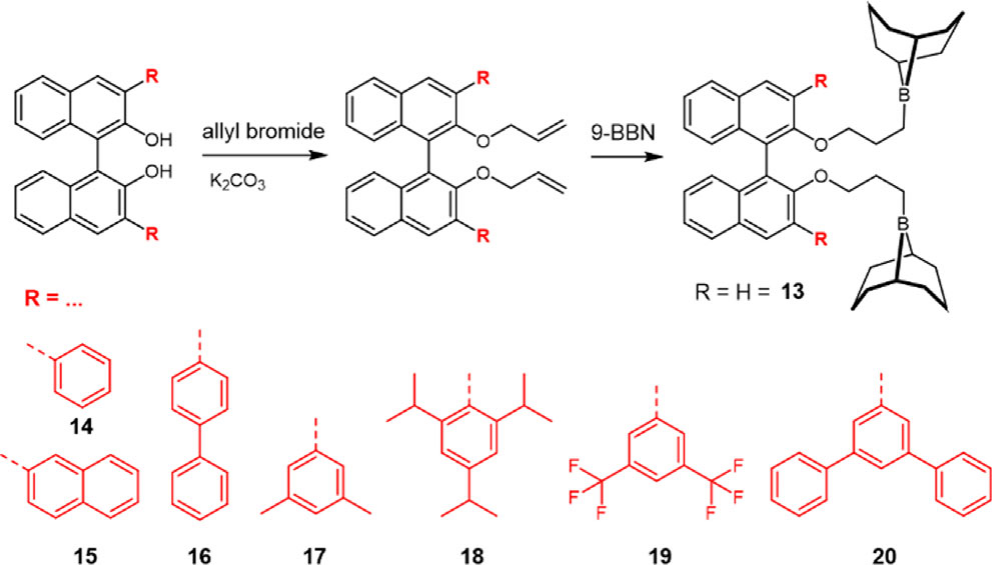
Preparative pathway to BINOL‐based chiral diboranes and compounds screened with 3,3^′^‐disubstitution patterns.

Naumann and coworker then installed a chiral, yet nonionic linker, based on the well‐available 1,1^′^‐bi‐2‐naphthol (BINOL) scaffold (**13**). The corresponding synthesis route (**Scheme** **9**) does not include complex purification and can be conducted on a multigram scale. Thus, (*R*)‐**13** was synthesized using the axially chiral (*R*)‐BINOL as the starting material. In a straightforward two‐step process, the hydroxyl groups of BINOL were first etherified using allyl bromide, and the resulting allyl groups were subsequently subjected to hydroboration with the commercially available 9‐borabicyclo[3.3.1]nonane (9‐BBN). Using (*R*)‐**13** in combination with an organobase and benzyl alcohol (BnOH) as an initiator, PO could be polymerized with a moderate degree of *isotactic* diad placement (*m*) of up to 69%. This could be further improved to 82% by conducting the reaction under more dilute conditions (toluene, 2 M). This latter behavior was attributed to a decrease in undesired, nonselective intermolecular propagation steps (involving two borane moieties from two catalyst molecules) at lower concentrations of the catalyst.^[^ ^64^ ^,^ ^78^ ^]^ All the while the polymerization still remained controllable with *M*_*n*_ > 100 kg mol^−1^ and *Ð*_M_ < 1.2 being achieved.

Different organobases, including 1,8‐diazabicyclo[5.4.0]undec‐7‐ene (DBU), phosphazene bases, and *N*‐heterocyclic olefins (NHOs) were tested; however, they did not show a significant influence on polymer tacticity. Using enantiopure PO, it was found that (*R*)‐**13** was selective for (*S*)‐PO, whereby a selectivity factor of *s* = 8 was observed under bulk conditions, and *s* = 18 under dilute conditions (both at RT).

Kinetic studies further revealed a zero‐order dependence on monomer concentration, consistent with the proposed mechanism, in which the propagation step occurs only with PO activated by the borane catalyst, the concentration of which remains constant. The selectivity of the catalyst could be further enhanced by introducing phenyl substituents at the 3,3^′^‐positions of the BINOL backbone. (*R*)‐**14** could polymerize PO with an isotacticity (*m*) of up to 88% (THF, 2 M, rt). However, its structural modification came at the cost of significantly reduced polymerization rates compared to (*R*)‐**13**.

In a follow‐up study, the group of Naumann further investigated the influence of sterically demanding substituents at the 3,3′‐positions of the BINOL backbone.^[^ ^79^ ^]^ The synthesis of the modified catalysts (**15–** **20**) remained straightforward, as all further substituted BINOL derivatives were commercially available. The study revealed that, in particular, *meta*‐substituted aryl groups at these positions led to a relevant enhancement in the catalyst selectivity. Notably, catalyst (*S*)‐**19** proved especially effective, yielding PPO with an *isotactic* diad content of 92% (−36 °C, THF, 2M, *mm* = 81%), which, to the best knowledge of the authors, represents the highest selectivity reported so far for an organocatalyst in the synthesis of *it*‐PPO. The semicrystalline resulting materials displayed melting temperatures > 50 °C. Kinetic experiments using enantiopure PO revealed a selectivity factor of *s* = 35 in favor of (*S*)‐PO under the same conditions. Running this reaction at room temperature, selectivity drops to *s* = 6, while the rate of polymerization increases more than fivefold. When compared to the bimetallic catalyst system reported by Coates and coworkers, which features selectivity factors exceeding 100,^[^ ^48^ ^]^ it becomes evident that the selectivity of the present generation of organocatalysts still has considerable room for improvement.

**Figure 6:**
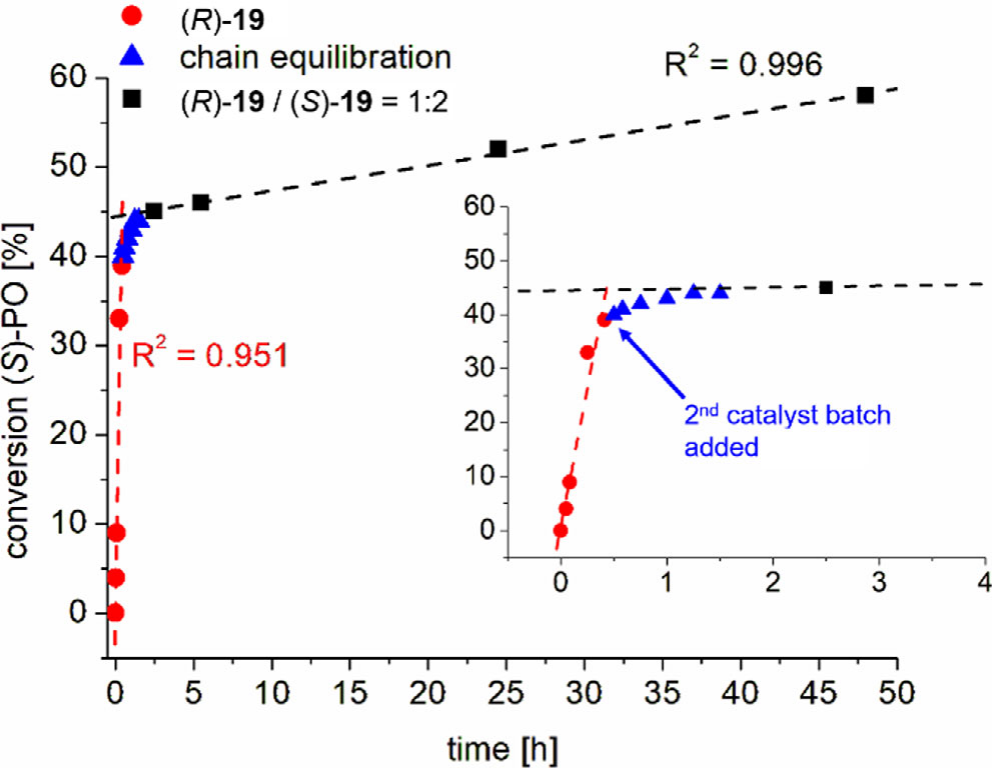
Polymerization kinetics using enantiopure PO and a first catalyst batch preferring this enantiomer, followed by a second catalyst batch with opposite preferences. Reproduced with permission.^[^ ^79^ ^]^ Copyright 2023, Royal Society of Chemistry.

When a *racemic* mixture of the catalyst was used instead of its enantiopure form, the isotacticity of the resulting PPO decreased only slightly (82% for (*R*)‐**13** vs. 80% for (*rac*)‐**13** and 92% for (*S*)‐**19** vs. 90% for (*rac*)‐**19**) under pairwise identical conditions conditions.^[^ ^64^ ^,^ ^79^ ^]^ In principle, chain transfer between the two enantiomers of the catalyst should result in scrambling of stereochemical information. The minor drop in isotacticity observed is therefore most likely attributable to a significantly faster rate of chain propagation compared to chain transfer, leading to the formation of extended blocks of either mostly (*S*)‐ or (*R*)‐PO during polymerization (see also discussion on dichromium catalysts in section 4.1). To quantify this effect, a kinetic experiment was conducted in which a polymerization was initiated using (*R*)‐**19** and its preferred enantiomer, (*S*)‐PO (reaction conditions: *t*Bu‐P_2_/(*R*)‐**19**/BnOH/(*S*)‐PO = 1:2:2.5:1000, RT, 2M in THF, **Figure** **6**). After monitoring the reaction for 0.5 h, with a conversion of ca. 40%, four eq. of (*S*)‐**19** were added. This addition was expected to significantly slow down the reaction once the growing polymer chains had equilibrated between the two catalyst populations, since (*S*)‐**19** is not the preferred catalyst for (*S*)‐PO. Indeed, following the addition, the reaction exhibited nonlinear kinetics for approximately one hour, likely corresponding to the time required for equilibration between the two catalyst batches. Thereafter, the reaction proceeded again with zero‐order kinetics, but at a noticeably reduced rate compared to before the second batch of catalyst was added.^[^ ^79^ ^]^

**Figure 7:**
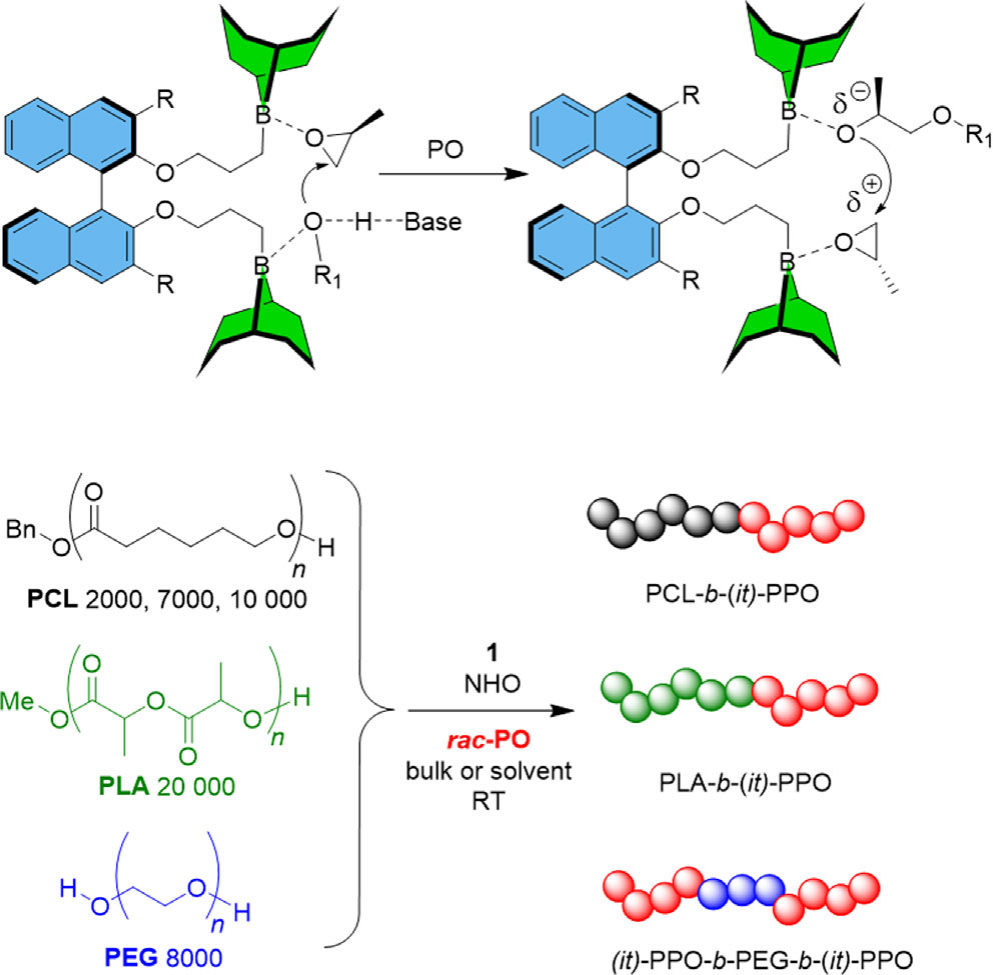
Top: Simplified cooperative action of a diborane catalyst in the presence of organobase, PO, and propagating chain end (R_1_‐OH). Bottom: Block copolymers with moderately *it*‐enriched polyether blocks. Reproduced with permission.^[^ ^64^ ^]^ Copyright 2022, Royal Society of Chemistry.

The use of borane catalysis also enables the incorporation of *it*‐PPO blocks into more complex polymer architectures. Traditionally, block copolymers of PO with polyesters are synthesized via a "polyether‐first" approach, as the harsh conditions typically required for conventional polyether synthesis can lead to degradation of the polyester segment. However, borane‐based catalyst systems have demonstrated the ability to reverse this strategy, enabling a "polyester‐first" route for *atactic* PPO, as demonstrated by Zhao and Ling, using triethylborane.^[^ ^80^ ^]^ Building on this concept, Naumann and coworkers demonstrated that the chiral diborane catalyst (*R*)‐**13** is capable of polymerizing *it*‐enriched PPO blocks onto both commercially available and custom‐synthesized poly(*ε*‐caprolactone) (PCL) and polylactide (PLA), allowing for the preparation of both di‐ and triblock copolymers (**Figure** **7**). Notably, even prolonged exposure (24 h) of PCL in THF to an NHO superbase in the presence of (*R*)‐**13** and BnOH did not result in any detectable polyester degradation.^[^ ^64^ ^]^

**Figure 8:**
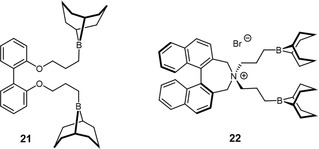
Left: Biphenyl‐derived catalyst (nonchiral analog to binaphthyl compounds), yielding *at*‐PPO. Right: Chiral spiro‐ammonium scaffold.

It should be noted that achiral control compounds, such as the biphenyl‐derivative **21** (**Figure** **8**), while highly active and robust in PO conversion,^[^ ^81^ ^]^ yield *at*‐PPO, in line with a selection mechanism that is catalyst‐controlled.

In a 2024 article, Wu and coworkers also conducted some investigations of different chiral borane catalyst systems with an emphasis on combining them with ammonium linked bifunctional moieties.^[^ ^82^ ^]^ Among the catalysts investigated were achirally linked diboranes with pinenyl‐derived borane moieties. However, these groups appeared to introduce substantial steric hindrance, ultimately suppressing polymerization under the applied conditions. Another evaluated catalyst motif (**22**), a bifunctional system based on a modified chiral spiro‐ammonium salt, had previously been investigated by Du, Lu, and coworkers in the copolymerization of CO_2_ with meso‐epoxides, where it achieved an enantiomeric ratio of up to 60:40 in the resulting polymer.^[^ ^83^ ^]^ In the homopolymerization of PO, **22** yielded an isotacticity (*m*) of 68%, which is slightly lower than that obtained with (*S*)‐**13** under comparable conditions. The authors attributed this moderate performance to the differing chiral architecture, with both borane moieties derived from the quaternary ammonium center, while the axially chiral unit remains relatively distant from the active site. They also examined the influence of different initiators on the isotacticity of the polymerization. Among those tested were PPN salts of various chiral and achiral carboxylates, as well as bromide salts of chiral and achiral quaternary ammonium compounds. When evaluated in combination with (*S*)‐**16** as the catalyst, none of the initiators led to a notable increase in the isotacticity of the resulting polymer. This observation is consistent with an enantiomorphic site‐controlled mechanism, in which the propagating chain end plays only a minor role in determining the stereoselectivity of the polymerization.

## 5 Applications of Stereocontrolled Polyethers

To date, there is no example of a commercialized product with tacticity‐controlled polyether components. This is certainly down to a lack of practicable, precise synthesis pathways in the past. Maybe more importantly, this signal lack of interest from large‐scale producers of polyethers can also be attributed to a long‐standing uncertainty about the potential benefits that could be gained from, e.g., *it*‐polyether materials.

Luckily, on both frontiers, the last ten years have seen very notable advances. As discussed above, catalyst design has focused on aspects of molar mass distribution and molar mass control, more efficient usage of *racemic* epoxide feedstock and functional group tolerance, all of which will increase the appeal of the corresponding stereoselective polymerization processes. Moreover, an increasing body of literature emerges which highlights the benefits of *it‐*polyethers as potential engineering plastics with advantageous properties (such as programable degradation or bio‐based building blocks) or as agents in self‐assembly and at interfaces.

**Figure 9:**
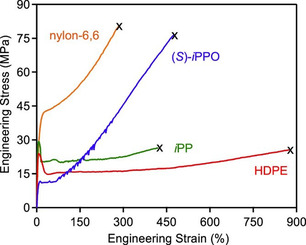
Stress–strain correlation of *it*‐PPO (*mm* > 96%) in comparison to frequently employed thermoplastic polymers. The serrated response observed for *it*‐PPO is an inherent property of the polyether material resulting from stress oscillations during measurement. Reproduced with permission.^[^ ^84^ ^]^ Copyright 2020, American Chemical Society.

An illustrative example was published in 2020, again by the Coates group.^[^ ^84^ ^]^ A series of stereocontrolled PPO samples was prepared by the application of dichromium catalyst (*S*)‐**8** or (*rac*)‐**8** in the presence of 1,6‐hexanediol as CTA and [PPN]Cl as cocatalyst. The thus received enantioenriched, enantiopure or *it*‐stereoblock semicrystalline PPO was then subjected to stress–strain investigations to illuminate the mechanical properties of the respective materials. All of these materials were found to display a pronounced strain‐hardening with ultimate tensile strengths comparable to polyamide 6,6 (**Figure** **9**). Interestingly, this evokes the possibility of obtaining a high‐strength material with the added benefit of being degradable under environmental conditions; under UV‐light (365 nm) the well‐known susceptibility of aliphatic polyethers for photolytic, radical‐mediated degradation also applies for the semicrystalline, *isotactic* PPO. This entails a rather rapid loss of molar mass after exposure of several days. Thereby, it also seems plausible that formation of ling‐lived small particles (microplastics) will not pose an issue.^[^ ^84^ ^]^ With the help of UV‐stabilizers, frequently employed additives used to disrupt the radical processes responsible for polymer chain degradation, the ”programing” of *it*‐PPO life times seems a promising perspective for this type of material, for example in a marine environment.^[^ ^85^ ^]^

**Scheme 10:**
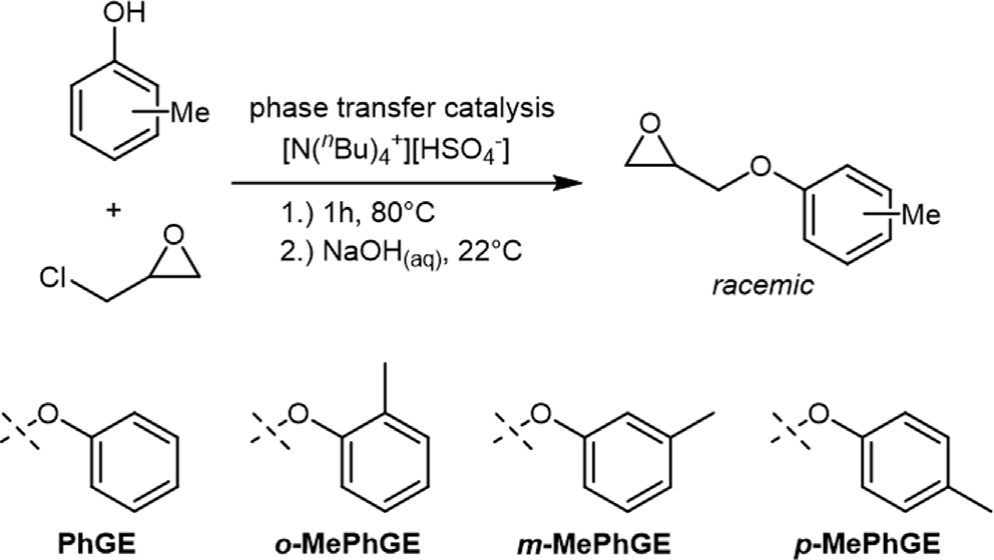
Preparation pathway for methyl‐substituted *rac*‐PhGEs.

While the relatively low melting temperature of *it*‐PPO (70 °C for an essentially stereoerror‐free sample with sufficiently high molar mass) puts an inherent limit to its applicability, other epoxide building blocks can be employed to yield a polymer with much higher *T*_m_. Very recently, in 2025, Frey and Coates published work on the stereoselective polymerization of PhGE and methyl‐substituted derivatives thereof (**Scheme** **10**).^[^ ^86^ ^]^ All monomers were prepared from bio‐renewable building blocks (substituted phenols and ECH can be sourced from lignin and glycerol, respectively) and subjected to polymerization via the Coates‐type catalyst **6** under typical reaction conditions (0 °C, ionic cocatalyst). Thereby, either (*rac*)‐**6** was employed, resulting in isoselective ROP, or (*S*)‐**6**, resulting in enantioselective ROP. Hence, in the latter case, one monomer enantiomer ((*S*)‐PhGE) was preferentially enchained, generating *it*‐poly((*S*)‐PhGE) and left‐over (*R*)‐PhGE. In the former case, both enantiomers were consumed with high selectivity, resulting in a *racemic* mixture of *it*‐poly((*S*)‐PhGE) and *it*‐poly((*R*)‐PhGE). Fittingly, using (*S*)‐**6**, conversion practically stops at around 50%, while application of (*rac*)‐**6** yields almost quantitative conversion. The levels of isotacticity were higher in the case of isoselective polymerization (*mm* > 92%) than in the case of enantioselective polymerization (*mm* > 78%). This follows from the fact that during enantioselective monomer enchainment, the undesired enantiomer steadily enriches in the feed, increasing the likelihood of stereoerrors. This again highlights the many advantages of making use of both monomer enantiomers, as also discussed in the previous sections. Analyzing the relative occurrence of stereoerrors, and in view of the absence of CTAs, the authors also could confidently exclude possible stereoblock formation (via chain‐shuttling when using (*rac*)‐**6**).^[^ ^86^ ^]^

With these polyethers in hand, the thermal properties of the different polymers were examined via DSC. For all four enantiopure polyethers, high melting temperatures were observed, ranging from *T*_m_ = 99 °C (*m*‐Me) to *T*_m_ = 185 °C (*o*‐Me). Strikingly, the *isotactic* racemates, resulting from the action of (*rac*)‐**6**, showed higher melting temperatures in all cases (alongside increased melting enthalpies). The size of the effect depended on the substitution pattern and was most pronounced for *m*‐Me (*T*_m_ increased to 175 °C). The overall highest melting temperature was identified for *p*‐Me and the parent unmodified PhGE with *T*_m_ = 214 °C and 216 °C, respectively. Overall, this behavior was attributed to stereocomplexation, whereby polymer chains with opposite stereoconfiguration can cocrystallize more effectively, pushing up the melting temperature. While this effect is well‐described for, e.g., poly(lactide), polyethers have been much less investigated for this useful effect. In fact, the findings summarized above constitute the first in‐situ polyether stereocomplex generation from *racemic* epoxide feedstock. Both the outstanding thermal properties as well as the bio‐based monomers render this work highly promising for future applications.

Amphiphilic polyether block copolymers are among the most frequently employed polymers for rheology‐control, drug delivery, hydrogel formation, or various forms of self‐assembly/structure‐direction. Very often, these come as triblock copolymers of the type PEO‐PPO‐PEO or PPO‐PEO‐PPO, which are also commercially available (*Pluronics* or *Reverse Pluronics*, respectively). For these substances, which are often applied at interfaces or for self‐assembly and micelle formation, the effect of replacing the typically *atactic* PPO segments by *isotactic* or *isotactic*‐enriched ones is essentially an open question and highly relevant in view of the large scale at which polyethers are employed in these areas.

**Scheme 11:**
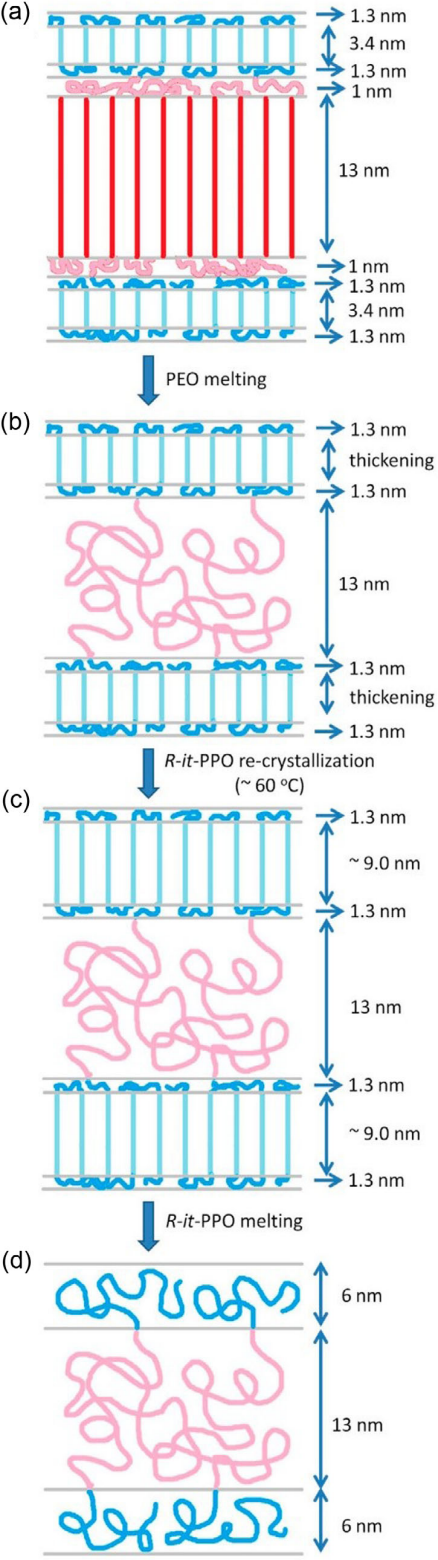
Melting sequence a,d) observed for (*R*)‐*it*‐PPO‐PEO‐(*R*)‐*it*‐PPO. The PEO melts first (a,b), followed by lamellar thickening c) of the *it*‐PPO segments. Reproduced with permission.^[^ ^87^ ^]^ Copyright 2015, American Chemical Society.

In a joint work, Kramer, Fredrickson, Hawker, and Lynd studied the phase transitions in (*R*)‐*it*‐*Reverse Pluronics* upon cooling or heating of the bulk block copolymer.^[^ ^87^ ^]^ Here, both segments can crystallize and the melting temperatures of the respective homopolymers are relatively close to each other. This leads to an interesting inversion in the sequence of crystallization versus melting: while the PEO‐blocks rapidly crystallize upon cooling (at ca. 50 °C), forming well‐defined lamellae under the given conditions, the PPO only subsequently and much more slowly then crystallizes between the already formed PEO‐layers (at about 40 °C). When slowly heated, however, the PEO crystalline layers melt first (ca. 60 °C), while the PPO layers at this temperature undergo lamellar thickening and pronounced recrystallization (**Scheme** **11**). The *it*‐PPO segments thus crystallize under hard confinement but melt under conditions of soft confinement. Perspectively, such research could open the way to improved nanostructured materials.

**Figure 10:**
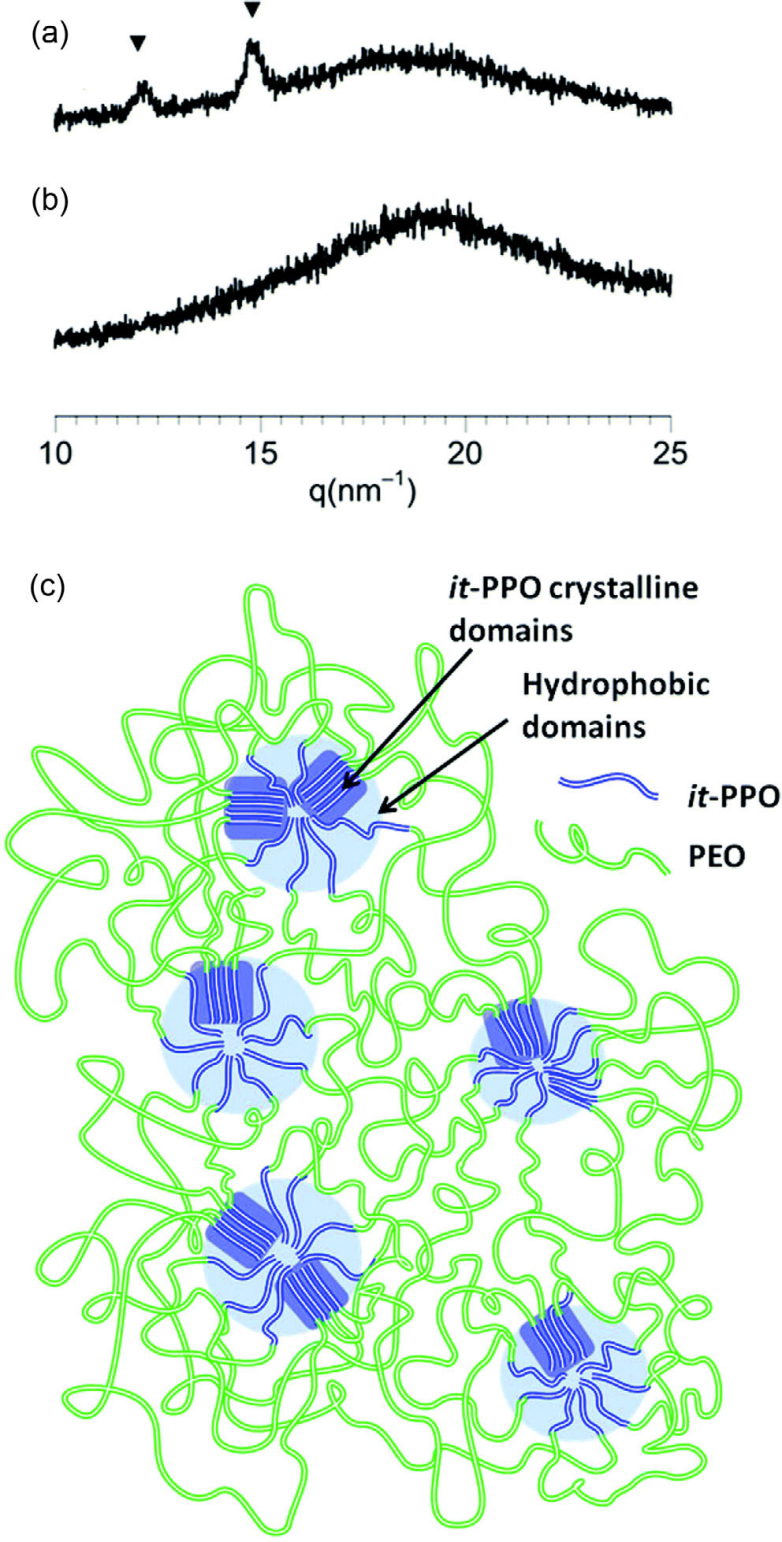
Top: Wide‐angle X‐ray scattering of a) hydrogel with *it*‐PPO crystallites and b) using *atactic* polyether. c) Bottom: Schematic representation of the semicrystalline *it*‐PPO‐PEO‐*it*‐PPO‐based hydrogel. Reproduced with permission.^[^ ^88^ ^]^ Copyright 2015,Royal Society of Chemistry.

In 2015, the groups of Lynd and Hawker successfully used the same type of polymer ((*R*)*‐it‐*PPO‐PEO‐(*R*)*‐it‐*PPO) to study hydrogel formation.^[^ ^88^ ^]^ The block copolyethers were prepared via anionic polymerization of the enantiopure PO (the latter obtained via hydrolytic kinetic resolution under application of a Co–Salen complex). This resulted in well‐defined amphiphilic block copolymers with *mm* > 90%, a sufficient level to enable crystallization of the PPO segments. A typical *Pluronic* with *at*‐PPO blocks is soluble in water at low temperatures but rapidly gels when the temperature increases to ambient or body temperatures (depending on polymer loading and polymer block lengths). This type of hydrogel is therefore suitable for, e.g., injectable gel systems in the medical context.^[^ ^89^ ^,^ ^90^ ^]^ The hydrogel resulting from the *Reverse Pluronics* polymer architecture is thereby stabilized by physical, reversible cross‐links formed via a micellar network (one PPO‐PEO‐PPO chain can connect two micelles). Interestingly, when (*R*)*‐it‐*PPO‐PEO‐(*R*)*‐it‐*PPO is hot‐pressed to a film and then placed in distilled water to swell, a hydrogel is formed in which the *it*‐PPO crystallites can still be observed (while the PEO has dissolved, see **Figure** **10**). Future applications and more complex architectures can be envisioned for such semicrystalline, polyether‐based hydrogels.

**Figure 11:**
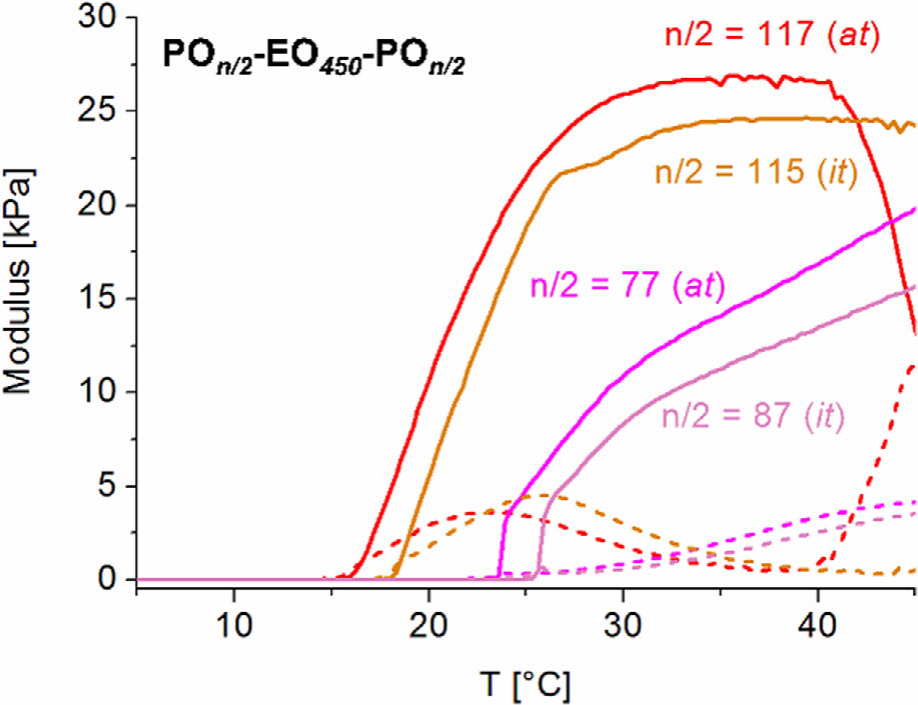
Comparison of hydrogel properties (15 wt.‐% polyether loading) resulting from *atactic* and moderately *isotactic*‐enriched (*mm* = 63%) PPO‐PEO‐PPO‐type polymers. Dashed line: loss modulus, full line: storage modulus. Reproduced with permission.^[^ ^91^ ^]^ Copyright 2020, Wiley.

In 2020, Naumann and coworkers investigated PPO‐PEO‐PPO‐type polymers for hydrogel formation, with a special emphasis on the impact of the respective block sizes on hydrogel properties.^[^ ^91^ ^]^ Part of this study also concerned *Reverse Pluronics* with PPO segments enriched to different levels of isotacticity. It was found that even moderately enriched samples behaved differently from fully *atactic* samples; consistently, gel temperatures were found to be higher and storage moduli lower for the isotactic enriched samples (**Figure** **11**).

**Scheme 12:**
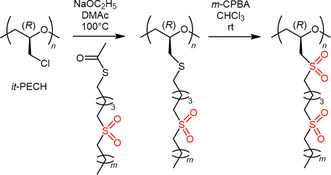
Comb‐like polymer with *it*‐polyether main chain and pendant groups containing sulfonyl moieties as synthesized by Zhao, Zhu, and Kwok.^[^ ^93^ ^]^ *m*‐CPBA =*meta*‐chloroperoxybenzoic acid.

In their quest for finding new polymers that display well‐defined ferroelectric liquid crystalline self‐assembly, Zhao, Zhu, Kwok, and coworkers have prepared comb‐like polymers based on *it*‐PECH with pendent groups containing sulfonyl functionalities.^[^ ^92^, ^93^, ^94^ ^–^ ^95^ ^]^ The *it*‐PECH was derived from an enantiopure monomer. The pendant groups were widely varied, for example, regarding the distance of the sulfonyl groups from the chiral main chain or the number of sulfonyl groups per side chain (**Scheme** **12**). The general rationale of this work was the avoidance of typical mesogenic groups (aromatics, long alkyl chains) and instead employ functionalities with high dipole–dipole interactions (sulfonyl) to achieve higher spontaneous polarization; at the same time, ready ferroelectric switching must still be able to occur, in sum imposing significant requirements on rational polymer design. The effect of the *isotactic* versus *atactic* comb polymer was typically the most pronounced when the sulfonyl groups were closely positioned to the main chiral centers. In these cases, *T*_m_ and the liquid crystalline transition temperatures were pushed much higher. ^[^ ^93^ ^]^ Depending on the composition of the pendant groups, different smectic (Sm) phases could be observed, for example, SmA, ^[^ ^92^ ^]^ SmA and SmE, ^[^ ^93^ ^]^ or SmA and SmC. ^[^ ^95^ ^]^ While further optimization is still necessary, in the future such *it*‐polyether‐based liquid–crystalline materials might find employment in electric and optical applications.

## 6 Summary and Outlook

In the past 10 years, significant advances have been achieved in the stereoselective polymerization of epoxide monomers. Research groups have mainly focused on improved control over polymer molar masses and their distribution, striving for well‐controlled polymerization reactions whereby, preferentially, chain‐transfer agents should be used to determine the degree of polymerization. This aspect is a key requirement not only for practicability, but also for larger scale production: polyalkoxylation reactors in general operate by dosing epoxide to a CTA/catalyst mixture, both to minimize dangers from the exothermic ring‐opening as well as to maximize control over the progress and rate of polymerization. Compatibilizing stereoselective polymerization and CTA usage is consequently a very good way of rendering a future stereocontrolled polyether product that much more likely. Scientifically, this also remains highly intriguing, since by its very nature the presence of CTAs entails an increased shuttling of polyether chains between catalysts. In the case of *racemic* catalyst mixtures and *racemic* epoxide monomers, this can lead to efficient feedstock conversion, rapid polymerization kinetics and excellent selectivity; however, the respective shuttling kinetics and propagation rates must fit, otherwise scrambled, *atactic* product will result.

Functional group tolerance, enabling, for example, the use of functionalized (macro)initiators, has been another research focus within the past decade. Progress in this field is especially important since it allows to go beyond *it*‐polyether homopolymers. Indeed, the emerging potential of block copolymers containing *it*‐polyether segments has been discussed in the above on several occasions.

Both metal‐based as well as metal‐free catalysts have contributed to these advances. Regarding organometallic approaches, the established Cotes‐type catalysts have been improved with the current generation of dichromium catalysts combining excellent stereoselectivity and high polymerization control. On the other hand, the relatively new chiral diborane motif has likewise emerged as a promising approach for stereoselective epoxide polymerization. Currently, the former display superior stereoselectivity, producing levels of isotacticity that cannot be matched by the available organocatalysts at the moment. The latter may be advantageo considering accessibility and ease of synthesis, as well as functional group tolerance.

Future developments, apart from the more obvious optimization of existing systems, could encompass the design of more rigid diborane structures. The necessary geometries (e.g., Lewis acid—Lewis acid distance) can be inferred from successful organometallic compounds. Moving from homobimetallic setups to heterobimetallic or otherwise multimetallic ones may be another promising approach.^[^ ^96^ ^]^ Studying N‐Al interactions^[^ ^97^ ^]^ or earth abundant elements could also prove to be highly rewarding.^[^ ^15^ ^]^ It should further be pointed out that studies regarding the biocompatibility of the catalysts or catalyst residues in the material have not been addressed yet in detail. In view of the employed compounds (e.g., chromium, boranes), such information would be very valuable.

A convincing push for further research regarding stereoselective epoxide polymerization could come from the increasingly numerous and attractive applications of the *isotactic*, aliphatic polyethers, as is also clear from patenting activity.^[^ ^85^ ^,^ ^98^ ^]^ It is exciting to see that such polyether materials could be both versatile thermoplastics, with desirable properties regarding degradability and sustainability, ^[^ ^84^ ^,^ ^86^ ^]^ as well as part of interface‐active/self‐assembling polymer structures. ^[^ ^87^ ^,^ ^91^ ^,^ ^92^ ^]^ Considering the vast application of polyether segments in all sorts of additives or in drug delivery, the identification of further intriguing applications seems just a matter of time.

## Conflict of Interest

The authors declare no conflict of interest.

## Author Contributions

**Teo Borst**: writing—original draft (equal). **Stefan Naumann**: supervision (lead); writing—original draft (equal); writing—review and editing (equal).
